# Investigation of the adsorption properties of gemcitabine anticancer drug with metal-doped boron nitride fullerenes as a drug-delivery carrier: a DFT study†

**DOI:** 10.1039/d1ra09319c

**Published:** 2022-01-20

**Authors:** Shamsa Bibi, Shafiq Ur-rehman, Laryeb Khalid, Ijaz Ahmad Bhatti, Haq Nawaz Bhatti, Javed Iqbal, Fu Quan Bai, Hong-Xing Zhang

**Affiliations:** Department of Chemistry, University of Agriculture Faisalabad 38000 Pakistan shamsa.shafiq@uaf.edu.pk shafiq.urrehman@uaf.edu.pk; Institute of Theoretical Chemistry and College of Chemistry, Jilin University Changchun 130000 China

## Abstract

Anticancer-drug delivery is now becoming a challenging approach for researchers as it allows controlled drug delivery near cancerous cells with minimized generic collection and the avoidance of secondary side effects. Hence in this work, the applications of nanostructures as anticancer drug-delivery carriers were widely investigated to target cancerous tissues. Based on DFT calculations, we investigated the transition metal-doped boron nitride nanostructure as a drug-delivery agent for the gemcitabine drug utilizing the B3LYP/6-31G (d, p) level of theory. In this research, the adsorption energy and electronic parameters of gemcitabine on the interaction with the metal-doped BN nanostructures were studied. It has been observed that metal doping significantly enhances the drug-delivery properties of BN nanostructures. Among the investigated nanostructures, Ni–BN has been found to be the most prominent nanostructure to transport gemcitabine with an elevated value of adsorption energy in both the gas phase (−45.79) and water media (−32.46). The interaction between gemcitabine and BN nanostructures was confirmed through frontier molecular orbitals and stabilization energy analysis. The fractional charge transfer, MEP, NCI, and NBO analyses exposed the charge transfer from drug molecule to the BN nanostructures. Transition density maps and UV-VIS spectra were also plotted to investigate the excited-state properties of the designed complexes. Thus, the present study provides an in-depth interaction mechanism of the gemcitabine drug with BN, which reveals that metal-doped BN nanostructures can be a favorable drug-delivery vehicle for the gemcitabine anticancer drug.

## Abstract 2

Anticancer-drug delivery is now becoming a challenging approach for researchers as it allows controlled drug delivery near cancerous cells with minimized generic collection and the avoidance of secondary side effects.

## Graphical abstract

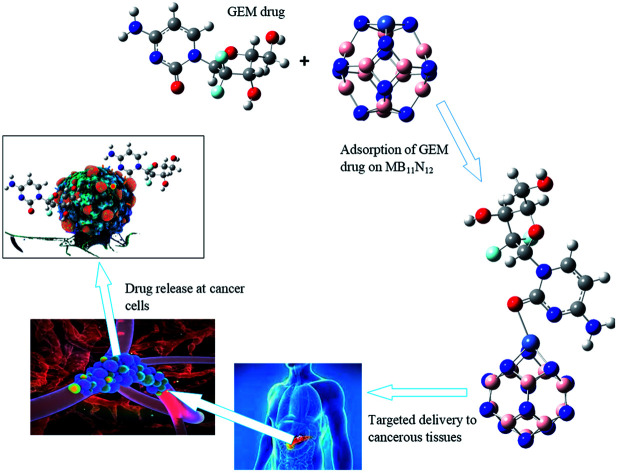

## 1. Introduction

Extensive research has been carried out not only to design anticancer drugs but also to develop new techniques for controlled drug delivery to the targeted site. Chemotherapy, radiation therapy, and immunotherapy are the ultimate solution to cure aggressive cancer. However, the main issue is the non-selectivity of numerous anticancer drugs that damage normal cells, such that their concentration that may even lead to toxic side effects.^1^ Therefore, it is necessary to deliver the precise concentration of the drug at a specific effective site for the required period. The efficient drug delivery to the targeted cells is a challenging approach that has triggered extensive exploration in the development of anticancer drug-delivery carriers.^2^

A wide range of drug-delivery vehicles are used to transfer drugs but the introduction of nanoparticles led to a great revolution which opened up a new horizon for the targeted drug delivery of anticancer drugs. These nanoparticles are adopted as effective drug-delivery carriers with a large surface area and small size which can easily penetrate the cell barriers.^3,4^ A series of nanostructures, including carbon nanotubes, fullerenes, and boron nitride fullerenes, have been studied computationally and experimentally to investigate their drug-delivery capabilities. The small size of these nanostructures allows them to easily conjugate with drug molecules.^5^ The surface properties and nontoxic nature of these nanostructures provide targeted delivery without affecting healthy cells, and therefore they are used as delivery vehicles.

In the extending field of nanotechnology, boron nitride (BN) nanostructures have attracted interest from researchers due to their exceptional physicochemical properties which make them advantageous for utilizing as drug-delivery carriers.^6^ The inorganic structure of BN with elevated chemical and thermal stability, wide bandgap, and upright mechanical strength make them favorable nominees for utilizing in drug delivery.^7,8^ The nontoxic nature of BN fullerenes was reported in 2009, which make them a suitable candidates for applications in medical science.^9^ The stability of the BN nanostructure is due to the ionic bond between the positively charged B and negatively charged N atoms, which makes them able to bond with other molecules. These unique features of boron nitrite fullerenes have motivated scientists to investigate their applications in nanomedicine and biomedical science and also their interaction with drugs.^10^

BN fullerenes have high sensitivity and reactivity as compared to carbon-based fullerenes and their reactivity can be enhanced through surface modulation. There are several types of fullerene-like BN nanostructures, such as B_10_N_10_, B_12_N_12_, B_24_N_24_, and B_28_N_28_, but B_12_N_12_ is the most stable structure compared to the others.^10–12^ Their optical, electronic, and chemical properties can be significantly enhanced through the adsorption and doping of certain metal atoms, which make them an ideal candidate for drug-delivery applications. Recently a DFT investigation was performed to study the adsorption of ifosamide on B_12_N_12_, which reported the suitability of the BN nanocage for drug delivery.^13^ The interaction of 5-fluorouracil,^14^ metformin,^15^ 5-aminolevulinic,^16^ and aspirin^17^ with B_12_N_12_ was also studied through a computational approach. The adsorption behavior of the lomustine anticancer drug on interaction with B_12_N_12_, B_24_N_24_, Al-doped B_24_N_24_, and Ga-doped B_24_N_24_ was explored theoretically and it was concluded that the modification through doping enhanced the adsorption property and might be a better choice for drug transport.^18^ Gemcitabine (GEM) is a nucleoside analog and chemotherapeutic drug also known as (2′-deoxy-2′,2′-difluorocytidine monohydrochloride).^19^ It was originally investigated as an antiviral drug and now has been used for various cancers as anticancer therapy. GEM as a prodrug converts into active metabolites that act by replacing the nucleic acid building blocks during DNA elongation, inhibiting the tumor growth and promoting apoptosis, which results in chain termination.^20^ It shows a cytotoxic effect against a wide range of tumor cells and inhibits their growth. The structure and mechanism of action are similar to cytarabine but it has a broader antitumor activity spectrum.^21^

In a comparative study for advanced pancreatic cancer, GEM was proved to be more effective than fluorouracil concerning the clinical status and survival rate. It shows activity against various tumors and has been approved for the treatment of the pancreas, lungs, ovaries, and breast cancer.^22,23^ The low toxicity effect makes it an ideal candidate for the treatment of elderly and unfit patients. Due to the synergistic effect when combined with various chemotherapeutic drugs, most commonly cisplatin, it is used for the treatment of solid cancer.^21^ It is the first line of chemotherapeutic drugs for pancreatic cancer. However, its effectiveness is limited by its toxicity toward healthy cells and the induction of various side effects during treatment. To overcome these side effects, therapeutic agents are directly transported to specific effective sites with the employment of drug-delivery carriers in a controlled way for the required period. Hence, the delivering mechanism enables the drug to cross multistage biological barriers with minimized generic collection and this cut offs its toxicity.^24^

**Scheme 1:**
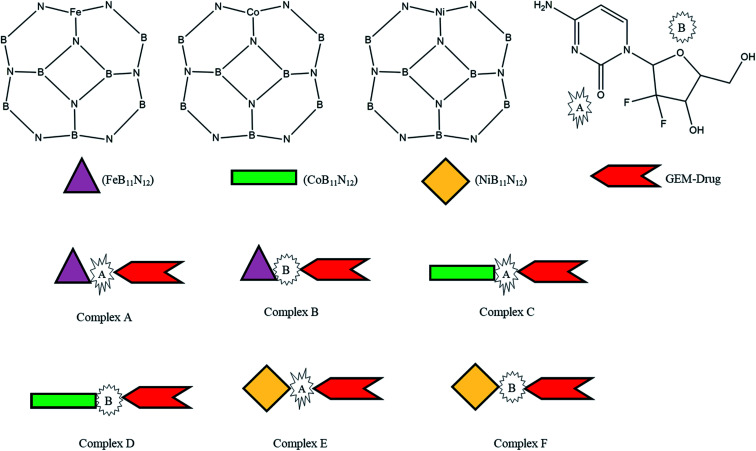
Scheme of the designed metal (M = Fe, Co, Ni)-doped boron nitride (BN) complexes.

Thus, inspired by the extraordinary properties and encouraging results of BN nanostructures, we attempted to explore the drug-delivery capability of transition metal-doped B_11_N_12_ for GEM anticancer drugs. In the present research, the interaction of the GEM drug through the oxygen atom of the pentagonal and hexagonal ring with metal-doped BN fullerene as the most active sites for interaction was studied through DFT calculations in the gas phase and water media. Moreover, we investigated three M [M = Fe, Co, Ni] doped B_11_N_12_ fullerenes to find suitable adsorbents for GEM drugs (Scheme 1). Notably, there is no theoretical study available on the interaction of GEM drugs with transition metal-doped BN fullerenes. The conclusions of this study may offer imperative perceptions for the design of experimental nanomaterial-based drug-delivery carriers. Some of the important electronic properties, such as the binding energy, solvation energy, bandgap, chemical hardness, and electrophilicity index, were calculated in this work. Furthermore, water media calculations were implemented to explore the solvent effect on the structure and electronic properties.

## 2. Computational details

For optimization, all the structures of molecules were designed through GaussView 06 to get the most stable conformation. Density functional theory (DFT) calculations were used to investigate the adsorption phenomenon and interaction probability between GEM drug and metal-doped BN. The optimization and energy calculations of different configurations of the drug and metal-doped BN were carried out by utilizing the B3LYP functional and 6-31G (d, p) basis set with the Gaussian 09 program.^25^ Frequency calculations were also carried out to verify that all the optimized structures were at the local minima with no imaginary frequency. The relative stability of the drug and nanostructure complexes were investigated based on the adsorption or binding energy. The adsorption energy (*E*_ads_) of the complexes was calculated through the following equation.^26^

1 *E*_ads_ = *E*_complex_ − (*E*_nanocage_ + *E*_drug_)

In the above equation, *E*_complex_ is the total energy of the drug attached to the metal-doped nanocage, while *E*_drug_ is the energy of the drug and *E*_nanocage_ is the energy of the metal-doped BN fullerene, respectively. The counterpoise (cp) correction method was used to eliminate the basis set superposition error (BSSE). The energy gap (*E*_g_) was also calculated to estimate the electronic stability. *E*_g_ is the energy distinction between the highest occupied molecular orbital (HOMO) and the lowest unoccupied molecular orbital (LUMO). The electrophilicity index (*ω*) is the main parameter to measure the energy stabilization when a system gets extra charge from the surrounding.^27^ The quantum mechanical signifiers, electronic chemical potential (*μ*), and chemical hardness (*η*) were also calculated from the following formula.^13^

2 *μ* = *E*_HOMO_ + *E*_LUMO_/2

3 *η* = −*E*_HOMO_ − (−*E*_LUMO_)/2

4 *ω* = *μ*^2^/2*η*

The kinetic aspects and thermodynamics of the interaction of the drug (donor) and BN nanostructures (acceptor) were studied. The GEM–BN interactions were evaluated using Δ*N* parameter, which indicated the number of electrons transferring from the donor to the acceptor. The Δ*N* factor can be computed as:5
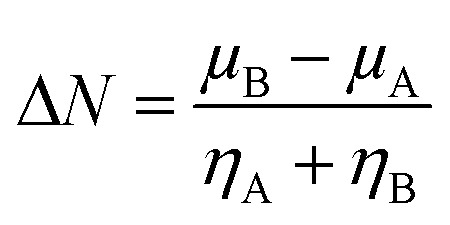
In the above equation, *μ* and *η* are the chemical potential and hardness of the donor (A) and acceptor (B), respectively. The negative values of Δ*N* indicate charge was transferred from the donor to the acceptor, while positive values indicate that charge was transferred from the acceptor to the donor. A comprehensive analysis of the stabilization energy (SE) was proposed and it was found that SE and charge transfer, as well as its different components, could be utilized to find the most stable non-covalent interactions.^28,29^ The structure stability, change in the energy of specie A (Δ*E*_A(B)_), and change in energy of specie B (Δ*E*_B(A)_), and overall stabilization energy Δ*E*_SE(AB)_ were calculated by the following formulas.6
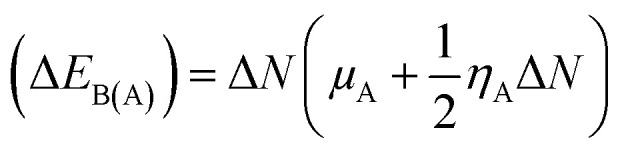
7
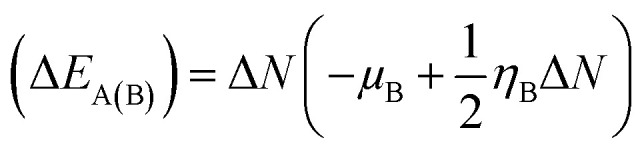

8 (Δ*E*_SE_) = (Δ*E*_B(A)_) + (Δ*E*_A(B)_)

Time-dependent density functional theory (TD-DFT) was utilized to obtain the UV-Visible spectra with the polarizable continuum model (PCM) in water media with the B3LYP and 6-31G (d, p) level of theory.^30^ Solvation energy (*E*_sol_) was also calculated to understand the solvation effect on the electronic parameters and stabilities of the structures, which could be computed from geometric optimization in both the gas phase and water media.^31^ To understand the charge distribution on the drug and the metal-doped BN, molecular electrostatic potential maps (MEPs) were also examined. We used PyMOlyze 2.0 to outline the density of states (DOS) spectra, which is a powerful tool to describe the orbital composition.^32,33^ For the non-covalent interaction (NCI) visualization between the drug and adsorbent, the VMD program was used. Multiwfn^34^ software was used for the natural bond orbital (NBO) and transition density matrix (TDM) calculations.

## 3. Results and discussions

### 3.1. Optimized geometry

**Fig. 1:**
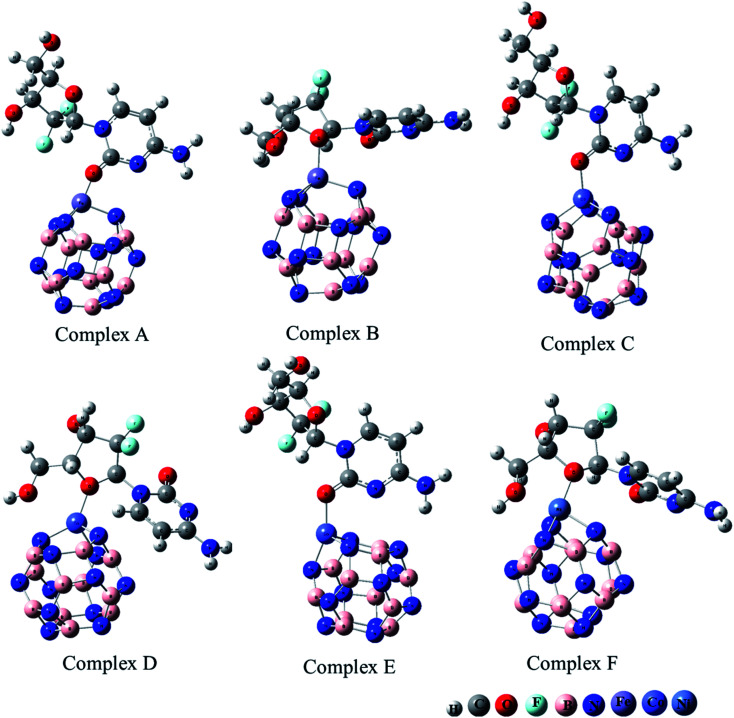
Optimized geometry of drug adsorbed on Fe (complex A, complex B), Co (complex C, Complex D), and Ni (complex E, complex F) metal-doped complexes calculated at the B3LYP/6-31G (d, p) level of theory.

The adsorption of GEM anticancer drug with metal (Fe, Co, Ni)-doped BN adsorbents was computationally studied to get higher stability conformations. The geometries of the GEM drug and metal-doped BN nanostructures were fully optimized utilizing the B3LYP/6-31G (d, p) level of theory (Fig. 1). The optimized nanostructure of pristine B_12_N_12_ consisted of eight identical hexagonal rings and six identical tetragonal rings, where nitrogen and boron atoms were placed periodically. Different bond lengths could be visualized between the B–N bonds of four and six-member rings.

In between two six-member rings, B–N bond length was 1.46 Å and between four and six members ring it was about 1.48 Å, which also coincides with the previous theoretical study of B_12_N_12_ nanocages.^35^ The substitution of one of the B atoms with Fe, Co, and Ni metal atoms produces the B_11_N_12_Fe, B_11_N_12_Co, and B_11_N_12_Ni complexes. After the doping of these metal atoms in the boron nitride nanocage, the geometry of B_11_N_12_M resembled that of the fullerenes. The structure was similar to the pristine BN nanocage, but its conformation was deformed. The doped metal atoms formed a covalent bond with N atoms and caused a considerable change in bond length. After the doping of Fe, Co, and Ni, the bond length between the nitrogen and dopant increased with the deformation in the resulting structures. Furthermore, the structural geometry of GEM before and after its adsorption to the metal-doped BN nanocage was analyzed. Though, no significant structural deformation was observed in the geometry of GEM after being absorbed onto the nanocage. Thus, the BN nanocage can act as a safe nanocarrier in drug-delivery systems for GEM drugs without degradation (Table 1).

**Table 1:** Optimized bond length (Å) of the investigated complexes

	Complex A	Complex B	Complex C	Complex D	Complex E	Complex F
M_24_–N_4_	2.01	1.84	1.86	1.88	1.86	1.89
M_24_–N_1_	1.81	1.79	1.87	1.89	1.90	1.91
M_24_–N_12_	1.82	1.85	1.83	1.84	1.89	1.87
M_24_–O_50_	1.93	—	1.95	—	2.01	—
C_29_–O_50_	1.26	—	1.26	—	1.23	—
O_32_–M_24_	—	2.13	—	2.11	—	2.14
N_11_–B_18_	1.48	1.47	1.48	1.48	1.48	1.47
N_12_–B_17_	1.48	1.48	1.46	1.45	1.46	1.46

### 3.2. Analysis of the adsorption energy

**Fig. 2:**
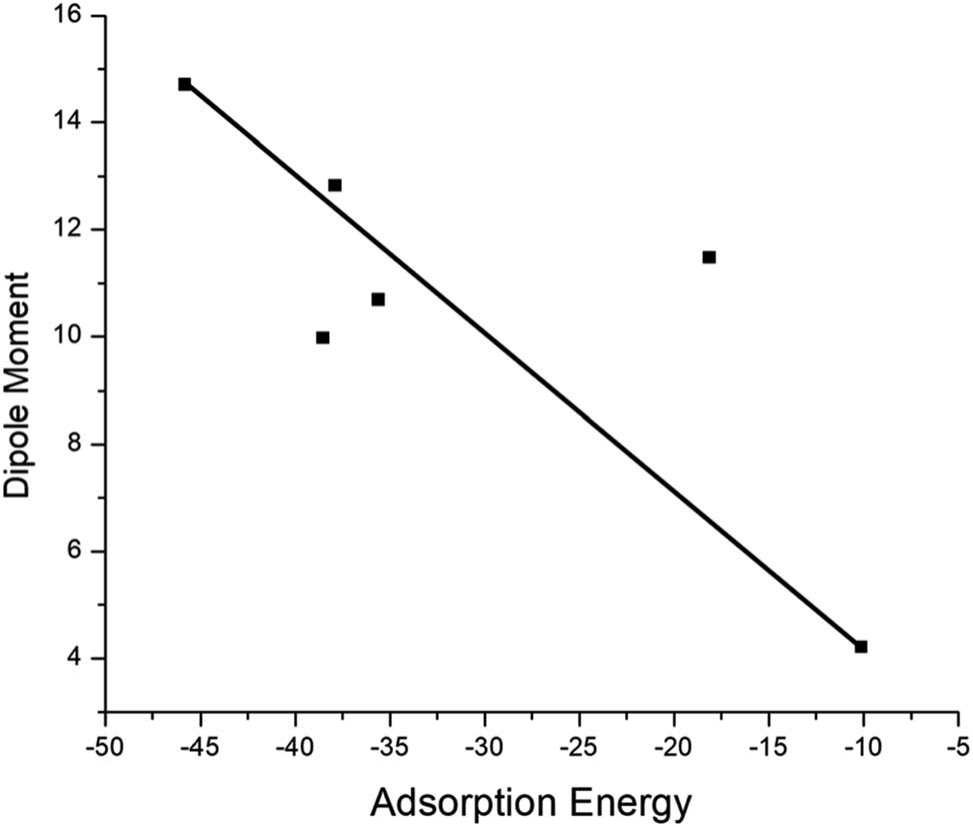
Graphical representation of the relationship between the adsorption energy and the dipole moment of metal-doped complexes.

The adsorption process of GEM drug and metal-doped boron nitride nanostructure was investigated through adsorption energy analysis, as given in Table 2. Following the analysis of the MEP of the drug and adsorbent, a drug mostly interacts through its O atom with the metal atom of the adsorbent. Depending on the different orientations of the GEM drug interaction with the metal-doped nanocage, these drug-delivery nanostructures were named as complex A, B for Fe–BN followed by complex C, D for Co–BN, and complex E, F for the Ni–BN nanostructure (Scheme 1). As predicted, the adsorption energy increased with the increase in the dipole moment and the drug molecules strongly interacted with the adsorbent. Subsequently, the strongest interaction of GEM was observed for Ni–B_11_N_12_ which had the highest dipole moment. The graphical representation of change in *E*_ad_ with the change in the dipole moment of the drug adsorbed on metal-doped BN nanostructures is also shown in Fig. 2.

**Table 2:** The adsorption energy (*E*_ad_), total energy in Hartree, and dipole moment values for all the investigated complex calculated in the gas phase utilizing the B3LYP functional and 6-31G (d, p) basis set

	Dipole moment (Debye)	Total energy (Hartree)	*E*_ad_ (kcal mol^−1^)
FeB_11_N_12_	0.30	−2195.3836	
Complex A	13.47	−3209.8257	−18.13
Complex B	4.20	−3209.8129	−10.14
CoB_11_N_12_	2.45	−2313.8451	
Complex C	14.25	−3328.3197	−38.53
Complex D	9.97	−3328.3186	−37.90
NiB_11_N_12_	2.02	−2439.3585	
Complex E	14.70	−3453.8447	−45.79
Complex F	10.69	−3453.8284	−35.60

The negative values of *E*_ad_ for all the designed drug-delivery complexes ensured the stable adsorption of different orientations of GEM drugs on the metal-doped BN nanostructures. The trend of *E*_ad_ was observed as: complex E > complex C > complex A with *E*_ad_ values of −45.79, −38.53, and −18.13 for the Ni, Co, and Fe complexes, respectively. However, besides the strong chemosorption of the GEM drug for the Ni-doped nanostructure, physisorption was also observed for other complexes with the decrease in negative value of *E*_ad_. The negative values of binding energy in both the gas and water phase proved the reaction feasibility between GEM drugs and the metal-doped BN nanostructure. After complete optimization, the intramolecular distance between the drug and metal atom was decreased, and complex E was found to give the most stable conformation as the distance between the drug and Ni atom was a minimum compared to the other complexes. Hence, all the metal-doped nanostructures with a negative value *E*_ad_ have the potential to be studied as anticancer drug-delivery carriers. Further, by comparing the doped BN nanostructures we can conclude that Ni-doped B_11_N_12_ showed a strong interaction for GEM drug with an elevated value of *E*_ad_ and Fe-doped B_11_N_12_ had the lowest interaction for GEM drug.

### 3.3. Molecular electrostatic potential maps (MEPs)

**Fig. 3:**
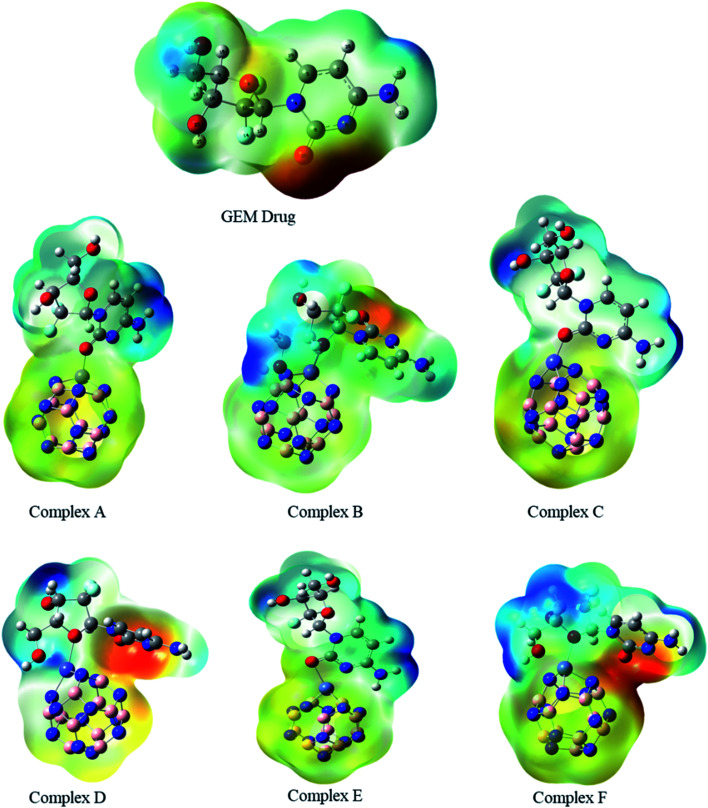
Molecular electrostatic potential maps of the investigated complexes. Blue to red color reveals the electron-deficient to an electron-rich regions of the surfaces.

The MEP map of the GEM was drawn to find the electronegativity and polarity of a drug molecule before the interaction of the drug and the BN nanocage. All possible adsorption sites between the drug and metal-doped BN could be observed utilizing MEP maps (Fig. 3). Two local minima were located at the oxygen of the hexagonal and pentagonal rings. This analysis also provides the electrophilicity and nucleophilicity characteristics of the drug molecule. Since the blue and red colors in the map show the most positive and negative sites, respectively, so oxygen atoms in the drug molecule, which are shown in red color, might be the most active sites for interaction with the metal-doped nanocage.^36^

MEP is an important parameter that indicates the net electrostatic potential at a certain point, generated through the total distribution of charge. Hence, based on ESP analysis, the doped metals can be expected to be the most active site for interaction of the drug with the BN nanocage, and so might be the most feasible region for adsorption. The drug molecule was placed at different orientations to get the lowest energy structure. The region around the molecular structure that is attractive for the positive charge is labeled as the negative electrostatic region with the higher charge density and the region that is repulsive for the positive charge is labeled as the positive electrostatic region. The MEP maps of the GEM drug and Fe, Co, and Ni-doped BN nanostructures can be observed in Fig. 3. Several negative electrostatic regions can be visualized in yellow and red color around the oxygen atom of the GEM drug, while blue color denotes the positive electrostatic region around the metal atom of the nanostructure adsorbent. After the interaction of the GEM drug with the metal atoms of B_11_N_12_, a negative charge is transferred from the drug to the adsorbent. It can be observed that this negative charge transfer from the drug to the adsorbent is more when a double-bonded oxygen atom of a respective drug is involved as compared to a single-bonded oxygen atom.

### 3.4. Electronic parameters

To investigate the electronic parameters of adsorbent molecules after the adsorption of a drug, DFT calculations were employed. The electrophilicity index is the main factor that increases with the increase in the electrophilicity of a system. It can be defined as a decrease in the energy of the system because of the extreme rate of electrons crossing the conduction band and provides perception about the stability, structural reactivity, and toxicity of chemical species. The values of *E*_HOMO_, *E*_LUMO_, and *E*_g_ are given in Table 3. It was observed for metal-doped complexes that the HOMO values decreased with the increase in LUMO values. However, after the adsorption of the drug, the HOMOs of the nanostructures adsorbent decreased while the LUMO values increased and the ionization potential also decreased, which allowed the electrons to move freely. These changes in *E*_HOMO_ and *E*_LUMO_ of the drug adsorbent led to a change in the *E*_g_ value. Compounds with a low *E*_g_ have a higher chemical reactivity and stability, which confirmed their semiconductor behavior. Due to slight changes in *E*_HOMO_ and *E*_LUMO_ of the investigated complexes after the adsorption of GEM drug, electronic parameters, such as the electrophilicity, softness, and hardness, were also altered slightly, and charge transferred between the drug and adsorbent occur, which indicated the ideal interaction between the drug and adsorbent. Moreover, the negative values of the chemical potential before and after the adsorption of the drug also indicated the stability of the investigated complexes.

**Table 3:** The *E*_HOMO_, *E*_LUMO_, energy gap (*E*_g_), chemical potential (*μ*), global hardness (*η*), ionization potential (*I*), and electron affinity (*A*) in the gas phase

	HOMO (eV)	LUMO (eV)	*E*_g_ (eV)	*I* (eV)	*A* (eV)	*μ* (eV)	*η* (eV)	*X* (eV)	*ω* (eV)
Drug	−6.40	−0.93	5.47	6.12	0.79	−3.45	2.66	3.45	2.36
FeB_11_N_12_	−7.12	−3.43	3.69	7.12	3.43	−5.27	1.84	5.27	7.54
Complex A	−5.85	−2.30	3.55	5.85	2.30	−4.07	1.77	4.07	4.68
Complex B	−6.39	−3.31	3.08	6.39	3.31	−4.85	1.54	4.85	7.64
CoB_11_N_12_	−7.15	−3.28	3.87	7.15	3.28	−5.21	1.94	5.21	6.70
Complex C	−5.81	−2.05	3.76	5.81	2.05	−3.93	1.88	3.93	4.11
Complex D	−6.19	−2.32	3.87	6.19	2.32	−4.25	1.93	4.25	4.68
NiB_11_N_12_	−6.84	−4.64	2.20	6.84	4.64	−5.73	1.10	5.73	13.10
Complex E	−5.74	−2.94	2.8	5.74	2.94	−4.34	1.40	4.34	6.73
Complex F	−5.84	−3.26	2.58	5.84	3.26	−4.55	1.29	4.55	8.02

The electrical conductivity of the investigated complexes was directly related to *E*_g_, as the materials with a decreased value of *E*_g_ have a higher ability to transfer electrons across the conduction band. Hence, materials with a low value of *E*_g_ show extreme electrical conductivity and *vice versa*. It can be observed from the values given in Table 3 that after the adsorption of GEM on the nanostructure, *E*_g_ decreased and a significant change in conductivity occurred. The hardness of a molecule is also an important parameter based on which the chemical reactivity can be observed. The molecules can easily transform the electron density and easily transfer their electrons from low excitation energy levels if they are structurally soft. After the adsorption of the drug to metal-doped BN, the chemical hardness of the drug was found to decrease, which promoted the adsorption processes and confirmed that the doping of Fe, Co, and Ni increased the solubility and polarity of the complexes. The hardness and *E*_g_ of the complexes were less than that of the GEM drug, hence the complexes were more polarizable and could accept electrons. Furthermore, it could be observed that the electronegativity value of the drug was less than that of the metal-doped adsorbent, so the electrons could be expected to flow from GEM to the metal-doped BN nanostructures.

The individual values of GEM and BN molecules, and change in energy of the donor (Δ*E*_A(B)_) and acceptor (Δ*E*_B(A)_) were calculated and are summarized in Table 4. The kinetic properties of the GEM drug adsorbed on the BN nanostructure were explored using (Δ*E*_A(B)_), (Δ*E*_B(A)_), and Δ*E*_SE(AB)_ to estimate the thermodynamic stability of the complexes. The GEM drug molecule was assumed to be a donor and the nanostructure adsorbent as an acceptor. The negative values of Δ*N* confirmed the electron transfer from the drug to the nanostructure adsorbent. However, the positive values of (Δ*E*_A(B)_) showed an energetically stable process in which electrons are transferred from the donor GEM drug molecule toward the acceptor BN nanostructures. In addition, the negative values of (Δ*E*_B(A)_) confirmed the stability of the complexes after their interaction with the drug. Finally, the negative values of Δ*E*_SE(AB)_ calculated from the equation confirmed the thermodynamic possibility of all the investigated complexes, while the most stable structure was complex E with Ni-metal doping on the adsorbent. This single-direction electron flow resulted in a significant change in polarizability after the adsorption of the drug with the metal-doped nanostructures. These investigations provide acceptable results about the interaction intensity of the drug, toxicity, such as protonation of the drug molecule in the low pH environment of cancerous cells, electrophilicity, and stability which could be employed in designing nanocarriers for a successful drug-delivery process to save or decrease the laboratory test duration.

**Table 4:** The fractional charge transfer (Δ*N*), energy change for the drug and adsorbent as Δ*E*_A(B)_ and Δ*E*_B(A),_ and the total energy change Δ*E*_SE(AB)_ (kcal mol^−1^) after the interaction of the drug with the adsorbent

	Δ*N*	Δ*E*_A(B)_	Δ*E*_B(A)_	Δ*E*_SE(AB)_
Fe-complex	−0.14	0.508	−0.553	−0.045
Co-complex	−0.11	0.395	−0.421	−0.026
Ni-complex	−0.21	0.783	−0.880	−0.097

### 3.5. Frontier molecular orbitals (FMOs)

**Fig. 4:**
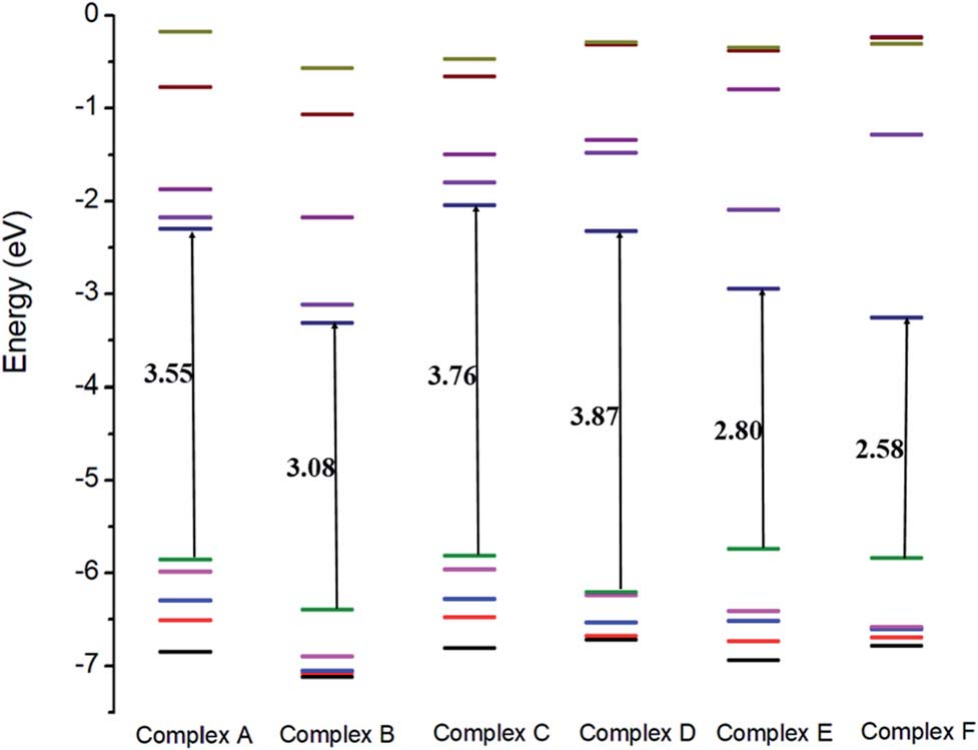
Comparison of the HOMO and LUMO energy levels for all the complexes.

**Fig. 5:**
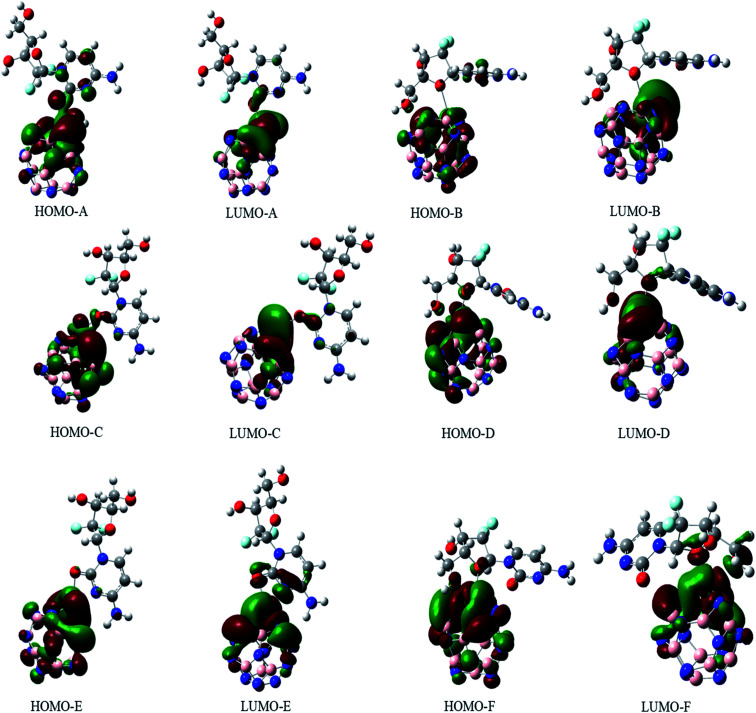
The HOMO and LUMO orbitals of all the investigated complexes.

The electronic properties of the GEM drug with metal doped adsorbent complexes were investigated through the HOMO, LUMO and FMOs of the complexes, as shown in Fig. 5. It could be observed that after the adsorption of a drug to the nanostructure, the HOMO energy level decreased while the LUMO energy level of the complexes increased, as shown in Fig. 4. The decrease in the HOMO energy level caused a decrease in the ionization energy in the adsorption of GEM drug and electrons could move spontaneously. The HOMO and LUMO orbitals were are uniformly distributed on the drug before its adsorption. However, their distribution was not uniform on the complex nanostructures after the adsorption of the drug, and the change in the distribution of orbitals was due to the charge transfer. For the complex, the HOMO was evenly distributed on the molecule, while the LUMO was on the GEM and some part was between the adsorbent and the drug. For the HOMO and LUMO, there was a large area of distribution between the GEM drug and metal-doped adsorbent, which indicated that chemical bonds were present between the molecules and their interaction supported the chemistry between them.

The sensitivity and reactivity of metal-doped adsorbent nanostructure were established from the FMOs and these led to the electron affinity (*A* = − *E*_LUMO_) and ionization potential (*I* = − *E*_HOMO_) calculations. Therefore, a decrease in HOMO energy caused a decrease in ionization potential and an increase in the reactivity of the complexes. The electron affinity of the complexes increased with the increase in *E*_LUMO_. The increase in the reactivity of the metal-doped complexes was also confirmed with the increased dipole moment. This significant increase in reactivity was observed for all the complexes, but the results with the Ni-doped complex were more prominent.

### 3.6. The density of states (DOS) analysis

**Fig. 6:**
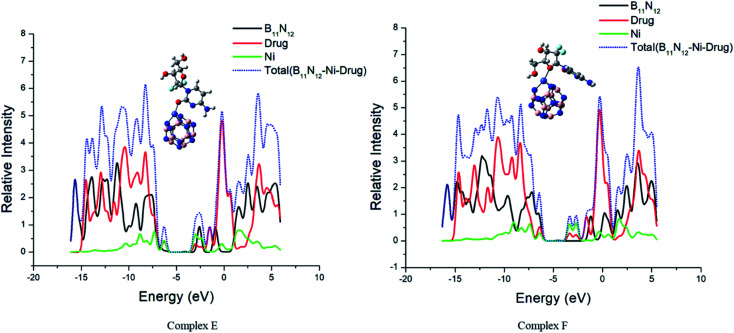
The calculated electronic density states of the TDOS and PDOS.

The interaction between GEM and metal-doped BN nanostructures was confirmed through the density of states (DOS) analysis given in Fig. 6 and S1.† From this analysis, a change in *E*_g_ for GEM adsorption on the different metal-doped complexes could be observed. DOS analysis defines the number of states for electrons available at different energy levels. A lower DOS value denotes the availability of a minimum number of states for occupation and a higher value shows a considerable availability of states for occupation. A considerable change in the *E*_g_ values was observed after the adsorption of GEM drugs on the metal-doped BN surface. By comparing the peak heights, it could be visualized that the total density of state (TDOS) was at a higher energy level than the partial density of state (PDOS), which shows a greater number of states were available for occupation. Also, no hybridization occurred between the TDOS and PDOS, which indicated a favorable adsorption of the drug for these complexes.

Notably, in the *E*_g_ range, electron excited states are not present and it is the range from the HOMO to LUMO that is related to the energy necessary for the outermost shell electrons to eject from the orbital and to become a moveable charge carrier in a matrix. Therefore, to determine the electrical conductivity of a system, *E*_g_ is a key factor. Our obtained result showed that after the interaction of GEM drug with the transition metal-doped BN nanostructures, a considerable decrease in *E*_g_ occurred, which shifted the behavior of the nanocarrier toward a semiconductor. It can be visualized from the PDOS analysis given in Fig. 6 that the main contribution to the HOMO was due to the drug molecules and the value of the LUMO was higher for metal-doped BN as they contributed to the adsorption process through the LUMO orbital. By comparing the LUMO values of different metal-doped complexes, it could be estimated that Ni metal highly contributed to the adsorption process and so resulted in an efficient electrochemical sensor.

### 3.7. Excited-state properties

**Fig. 7:**
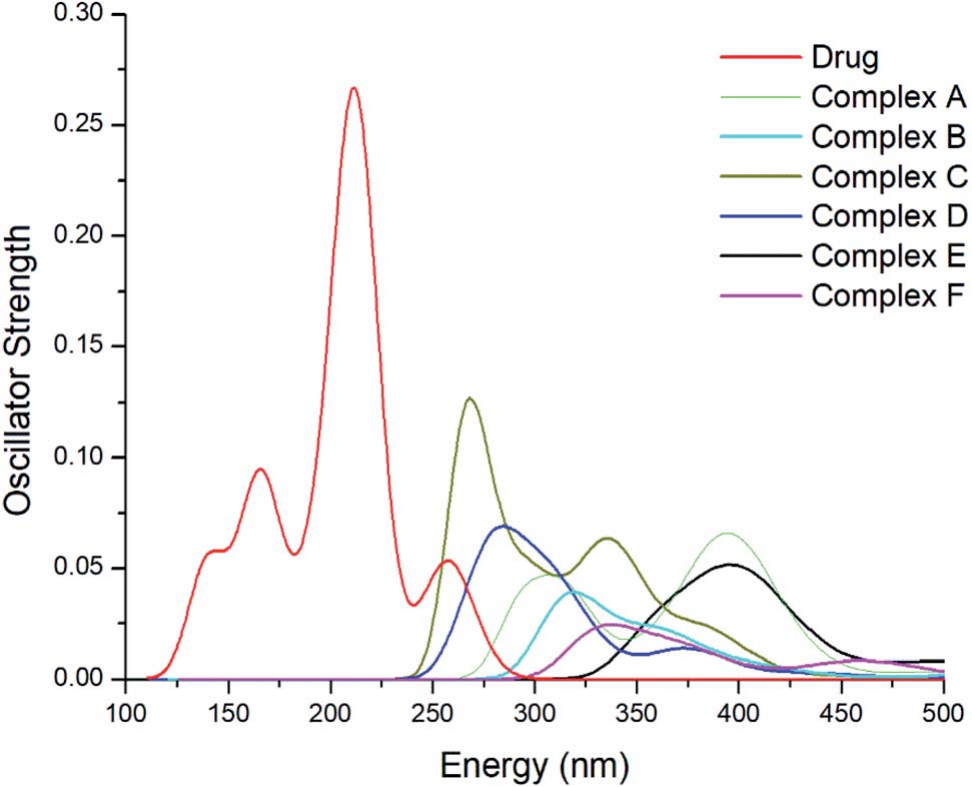
UV-Vis spectra of the GEM drug and all the metal-doped investigated complexes after the adsorption of the drug.

It is necessary to detect and monitor the concentration of the drug in a human body while developing an appropriate drug-delivery carrier for the successful delivery of anticancer drug molecules to reduce the toxic side effects to secondary cells. The concentration of GEM drug can be explored through higher performance liquid chromatography but before detection, complicated preparation is required through high-tech instrumentation. TD-DFT is an effective approach to study the parameters of a biological system. The online monitoring and instant detention of drug concentration might be possible through the UV-Vis spectral analysis of complexes. The excited-state properties of the drug and metal-doped complexes have been explored through an analysis of the UV-Vis spectra. The absorption spectra of the drug and metal-doped complexes after the adsorption of the drug were calculated theoretically and displayed in Fig. 7. It can be seen from spectra that the absorption peak of GEM drug was at 214 nm, while with the oscillator strength *f* = 0.27, the absorption values of the drug and all the investigated complexes were in a visible region ranging from 125–500. The absorption peak of the metal-doped complex was much lower compared to the drug before adsorption. The absorption peaks of the spectra were broadened and showed a red-shift after the adsorption of the drug onto the BN nanostructures and the peak was also weakened and broadened. This shift and weakness of the peak strength may be exploited for online observing the concentration of GEM drug in the body.

### 3.8. TDM and natural bond orbital (NBO) analysis

**Fig. 8:**
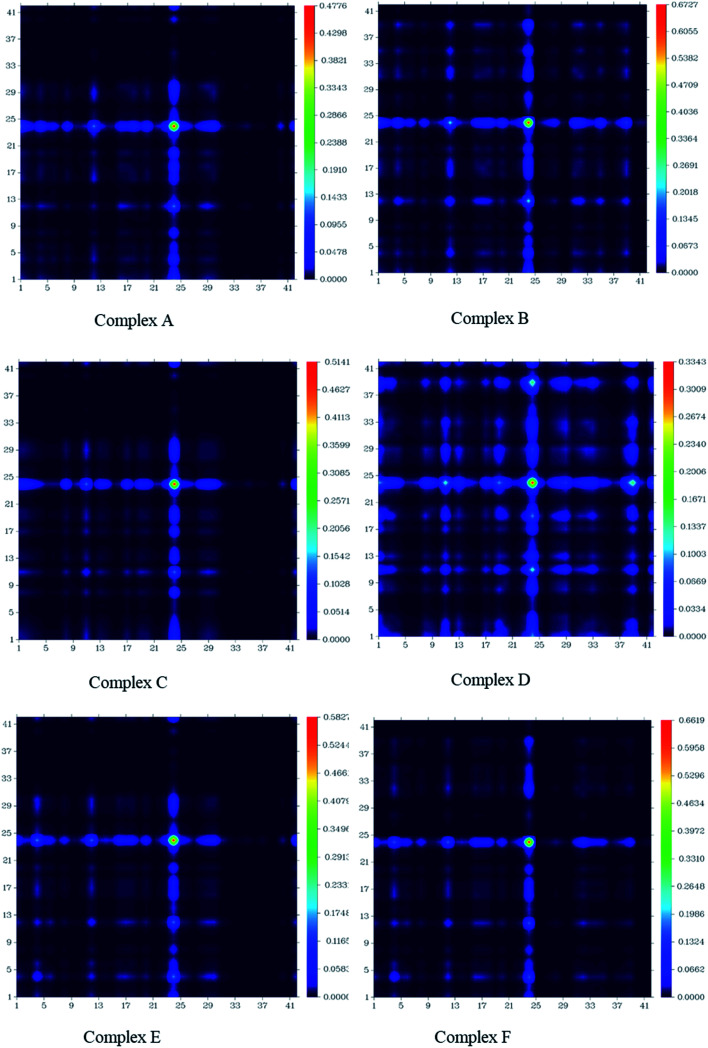
Transition density matrix of all the investigated complexes after the adsorption of GEM drug.

The charge transfer among GEM drugs and Fe, Co, and Ni-doped BN nanostructures during electronic excitation was estimated through a transition density matrix (TDM). The intramolecular charge transfer and magnitude of the transferred charge for all the complexes were plotted and quantified (Fig. 8).^37^ The *x*-axis represents the number of atoms and the right *y*-axis represents the electron density coefficients. TDM heat maps show the strong charge transfer in which every fragment of the complex is involved during electronic excitation. The region of density depletion and transitions involved in the excited states can be identified easily. It is obvious that there was charge transfer from the drug molecule to BN acceptor nanostructure from the S_0_ to S_1_ transition of all the designed compounds, therefore S_1_ is the predominant intramolecular charge transfer state, and the electrons were localized on the donor and acceptor parts. These results also following the FMOs given in Fig. 5. According to these results, it can be estimated that complex D had a larger charge transfer and the transferred charges of complex E and F were also larger. In fact, it can be seen from Fig. 8 that the concentration of charge transfer was in the middle of a complex between the drug and metal atom of the cage.

**Fig. 9:**
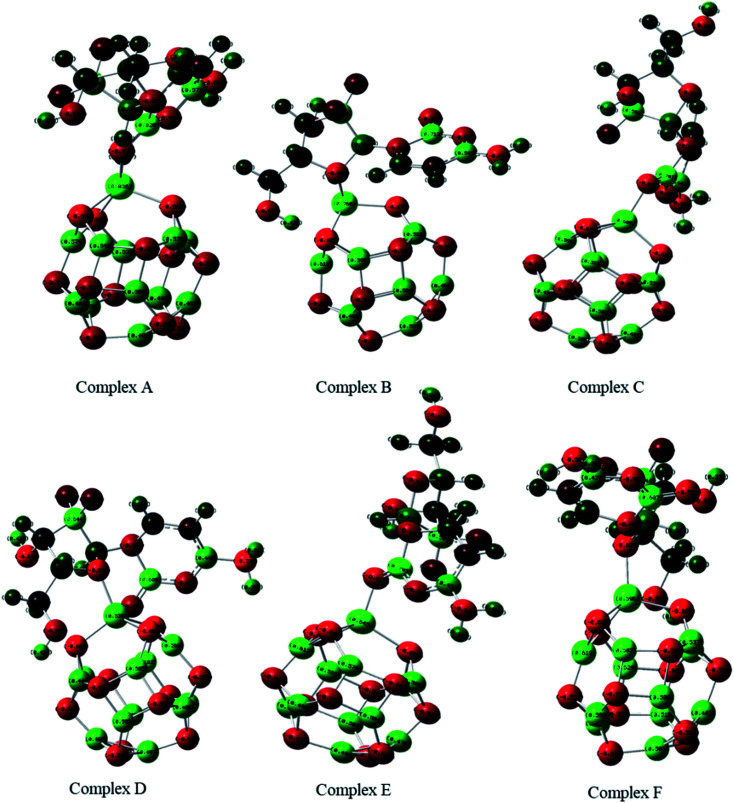
Natural bond orbitals analysis of Fe, Co, and Ni-doped BN complexes calculated through the B3LYP/6-31G(d, p) level of theory.

Furthermore, to estimate the electronic effect of GEM drugs on the BN surface and their properties as donor or acceptor around the adsorption position, a natural bond orbital (NBO) analysis was done. The interaction energy and second-order interaction energy between the bonding and antibonding orbitals were computed through NBO analysis.^38,39^9
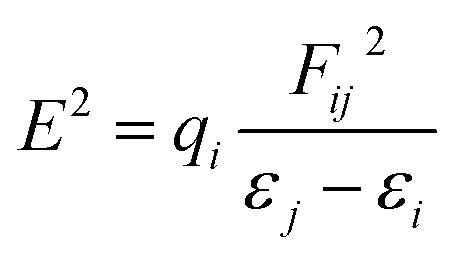
In the above equation, *q*_*i*_ and *F*_*ij*_^2^ are the donor orbital and off-diagonal occupancy, respectively, and *ε*_*i*_ and *ε*_*j*_ are the orbital energies of the designed complexes. Doping with Fe, Co, and Ni significantly increased the *E*^2^ value, which caused the electron density to transfer around the adsorption position and increased the adsorption energy. Furthermore, the direction of charge transfer was observed utilizing NBO analysis. The green and red colors in Fig. 9 denoted the positive and negative charges. The negative charge of the interacting oxygen atom of a drug decreased after its adsorption with the metal-doped BN nanostructures, which indicated charge transfer from the drug to the nanostructures.

### 3.9. Non-covalent interaction analysis (NCI)

**Fig. 10:**
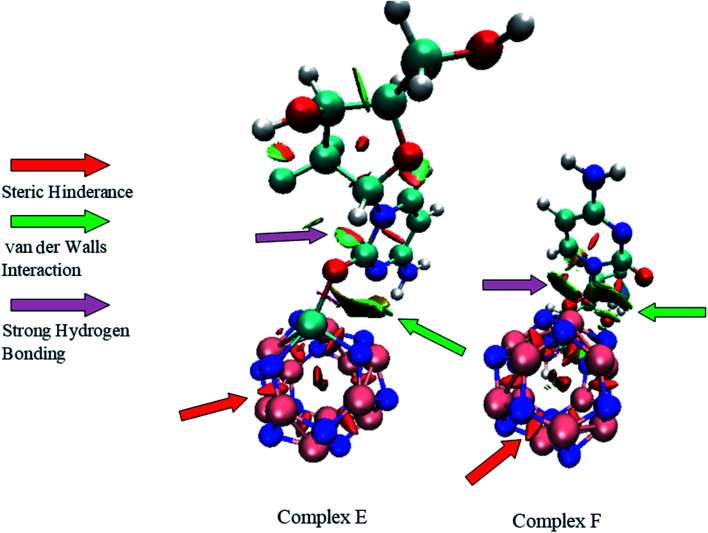
NCI plots of complex E and complex F.

In the field of drug designing, NCI analysis is an essential tool for various properties of biological systems. To understand the interactions between the GEM drug and metal-doped BN nanostructures, we performed NCI analysis based on the topological electron density. To detect the non-covalent attractive and repulsive interaction between the drug and nanostructures, NCI plots and reduced density gradient (RDG) analysis were performed. We used Multiwfn to perform the RDG analysis and for NCI visualization, the VMD program was used. RDG analysis also elucidates which interactions are stronger and which are weaker. In previous studies, it was exposed that weak interaction can be explained through the peak analysis that emerges at the low-density surface of reduced density maps.^40^ In NCI analysis, the self-consistent field (SCF) electronic density is investigated from the reduced density gradient and the peaks shown at low density are exploited to distinguish the mode of interactions. Electrostatic interactions or hydrogen bonding types as strong and attractive interactions are characterized with a blue color while a red color exhibits strong and repulsive steric interactions. The weak van der Waals interactions, such as H–π and π–π stack interactions, are categorized with a green color. The NCI plots of GEM drug adsorbed on the metal-doped BN nanostructures are shown in Fig. 10 and S3.† It can be visualized from the figure that the blue color near the hydrogen atom of the hydroxyl group indicates the interactions of hydrogen bonding. A larger green color patch between the drug and adsorbent formed on the interaction of the drug with the BN nanostructures can also be observed. Weak van der Waals interactions exist in these areas. Furthermore, red spikes appear as a red patch between B and N atoms, which can be recognized as steric repulsive interactions for our investigated complexes. Hence, van der Waals, H–π interactions, and hydrogen bonding also play an important role in the stability of drug-delivery complexes.

### 3.10. Solvent effect

To investigate the nature of the interaction of the metal-doped BN nanostructure with GEM drug for their application as a drug-delivery carrier, we optimized the nanostructure complexes in water solvent utilizing the polarizable continuum model (PCM). The calculated parameters of the investigated complexes are tabulated in Table 5. To study the solvent effect on the stability of the designed complexes, the solvation energy was computed through the following equation:

10 *E*_sol_ = *E*_water_ − *E*_gas_

Where *E*_water_ is the total energy of the complex in water media, while *E*_gas_ is the total energy in the gas phase. The solvation energy of GEM drug adsorbed on the metal-doped BN nanostructure was significantly increased, which confirmed the solubility in water media and successful delivery of the GEM drug to the cancerous cell environment. The negative value of solvation energy in water media also confirmed the stability of the investigated complexes. Furthermore, the solubility of the GEM drug in water media made the *E*_ad_ of our designed complexes less negative than their *E*_ad_ in the gaseous phase; this impact was also observed in previous work.^41^ Still, the maximum *E*_ad_ value was noted for complex E in which the drug was doped with Ni-doped metal atom. The dipole moment of the investigated complexes was significantly increased in water media. In the gas phase, the maximum value of the dipole moment was 15.47 and then followed by 14.82 and 14.70 for Fe, Co, and Ni-doped complexes, respectively. However, in water media, the values of the dipole moment rise to 21.49 for complex A, 20.79 for complex F, 20.75 for complex C, 20.42 for complex E, 16.93 for complex D, and 13.97 for complex B was observed. Hence, the elevated value of the dipole moment in water media confirmed the capability of the investigated complexes to dissolve in polar solvents with a significant increase in their reactivity, which is beneficial for GEM drug-delivery applications.

**Table 5:** The *E*_ads_, E_sol_, and dipole moment for all the investigated complexes in the water phase

	Dipole moment (Debye)	Energy (a.u)	*E*_ad_ (kcal mol^−1^)	*E*_sol_ (kcal mol^−1^)
FeB_11_N_12_	1.47	−2195.3957		
Complex A	19.49	−3209.8628	−17.80	−23.29
Complex B	13.97	−3209.8617	−17.14	−30.62
CoB_11_N_12_	7.48	−2313.880		
Complex C	20.15	−3328.3613	−26.21	−26.75
Complex D	16.93	−3328.3656	−28.94	−28.84
NiB_11_N_12_	6.83	−2439.3917		
Complex E	20.42	−3453.8822	−32.46	−23.55
Complex F	20.79	−3453.8734	−26.93	−28.20

## 4. Conclusion

In this study, the performance of metal-doped BN fullerenes (B_11_N_12_–Fe, B_11_N_12_–Co, B_11_N_12_–Ni) as drug-delivery carriers for the GEM anticancer drug was investigated utilizing DFT calculations. The interaction between the GEM anticancer drug and metal-doped BN nanostructure was investigated for the first time in both the gas phase and water media as comprehensive research. After optimization, the geometrical analysis showed that before and after the adsorption of the drug, the bond length between atoms was not significantly changed, which confirmed the stability. The metal-doped BN exhibited better reactivity with an elevated dipole moment and interacted more intensively with the GEM drug. The negative values of adsorption energy for all complexes in both the gas and aqueous phases expressed the favorable interactions between the BN nanostructure and the drug and the reaction was exothermic between them. The adsorption trend was observed as: complex E > complex F for Ni atom, followed by complex D > complex C for cobalt, and then complex A > complex B for the Fe-doped metal in the gas phase. Also, the results showed that doping with metal atoms, especially doping with Ni metal, improved the drug adsorption and it played a valuable role in the stability. It could also be seen from the results that in the gas phase, the adsorption process was more favorable and stable. A significant extent of charge transfer from GEM to the BN nanostructure was observed through fractional charge transfer and NBO analysis. The increased dipole moment in both the gas phase and water media confirmed the solubility of the investigated complexes in polar media, which is advantageous for drug-delivery applications in biological systems. The negative value of stabilization energy and solvation energy also confirmed the stability of the complexes. Finally, the Ni-doped complex E with the maximum stability and adsorption energy of −45.79 in the gas phase and −32.46 in an aqueous solution is considered an efficient candidate for the successful delivery of the GEM anticancer drug.

## Conflicts of interest

There are no conflicts to declare.

## Supplementary Material

RA-012-D1RA09319C-s001
