# Supplementary material for: Investigation of the adsorption properties of gemcitabine anticancer drug with metal-doped boron nitride fullerenes as a drug-delivery carrier: a DFT study
Source: RSC Adv. 2022 Jan 20;12(5):2873–87. doi: 10.1039/d1ra09319c (PMC8979131; doi:10.1039/d1ra09319c)
Supplement: RA-012-D1RA09319C-s001 [file RA-012-D1RA09319C-s001.pdf]

Electronic Supplementary Material (ESI) for RSC Advances.
This journal is © The Royal Society of Chemistry 2022

Supporting Information

Investigation of adsorption properties of gemcitabine anticancer drug with metal-doped boron nitride fullerenes as a drug delivery carrier; DFT Study.

*Shamsa Bibi *[1], Shafiq-Ur-rehman *[1], Laryeb Khalid [1], Ijaz Ahmad Bhatti [1], Haq Nawaz Bhatti[1], Javed Iqbal [1], Fu Quan Bai [2]. Zhang Hong Xing [2]*

[1]Department of Chemistry, University of Agriculture Faisalabad, Faisalabad 38000, Pakistan.

[2]Institute of Theoretical Chemistry and College of Chemistry, Jilin University, Changchun, 130000, China.

Corresponding authors*

shamsa.shafiq@uaf.edu.pk

shafiq.urrehman@uaf.edu.pk

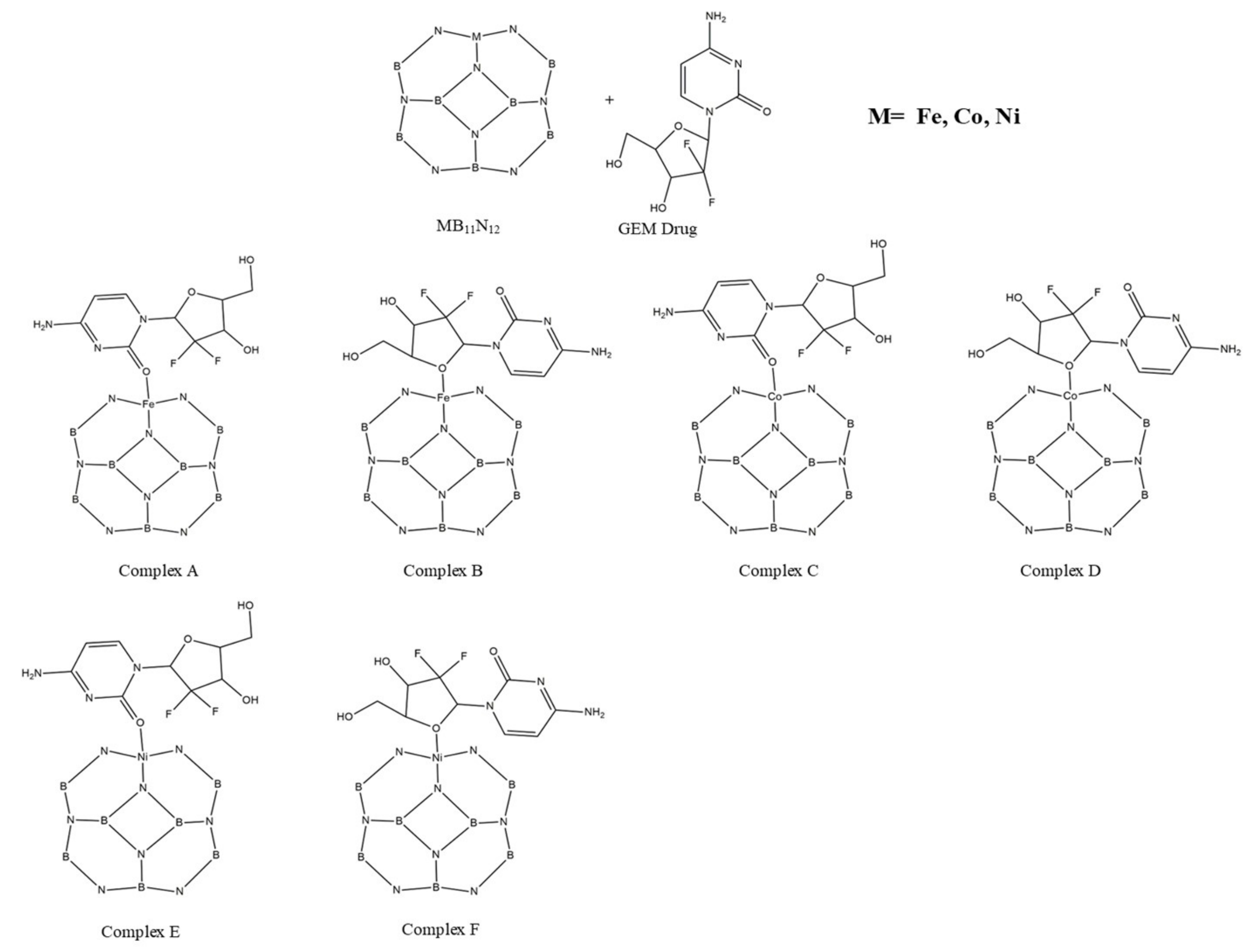

Scheme S1: Scheme of metal doped complexes ($CoB_{11}N_{12}$, $FeB_{11}N_{12}$, and $NiB_{11}N_{12}$) after interaction of GEM drug

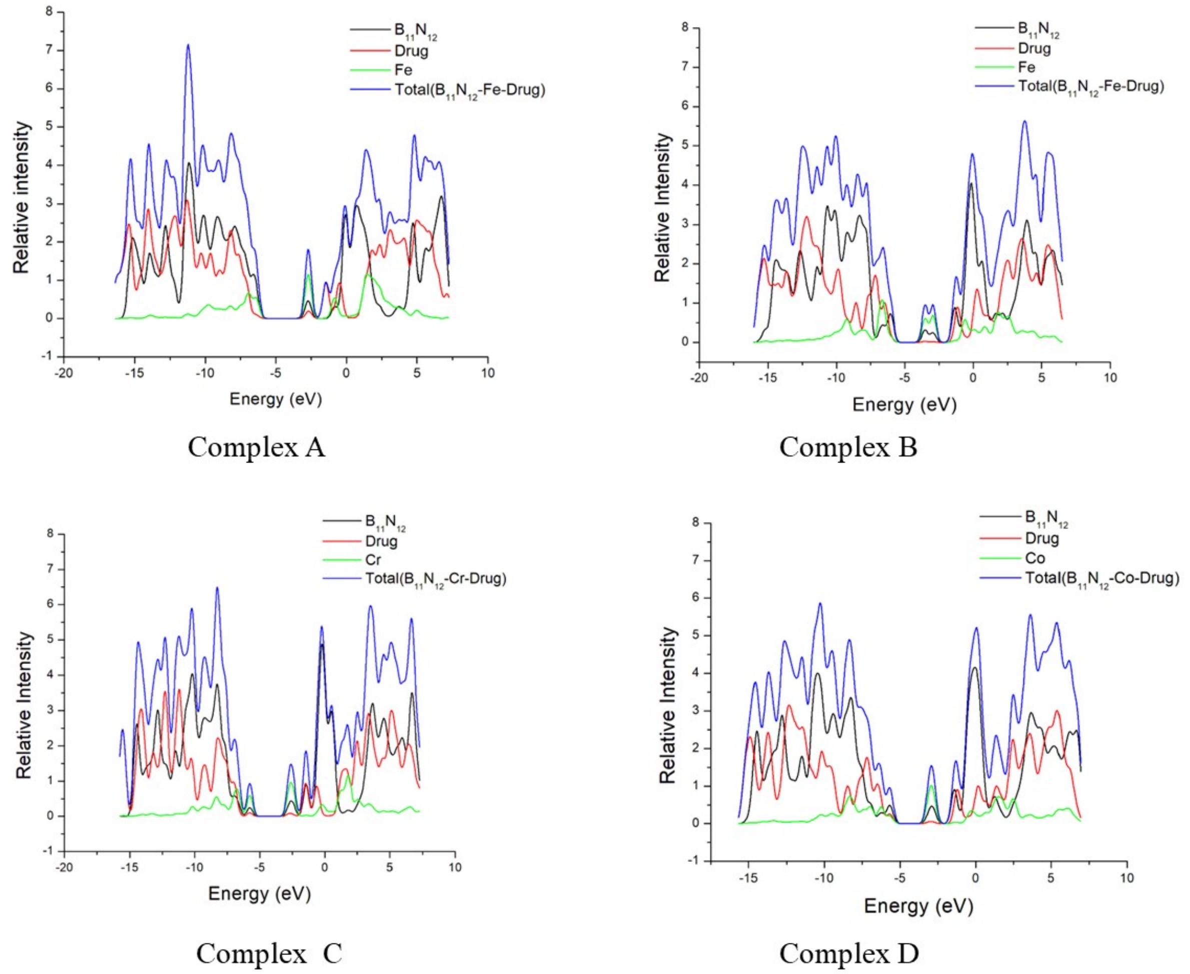

Figure S1: DOS spectra of investigated complexes.

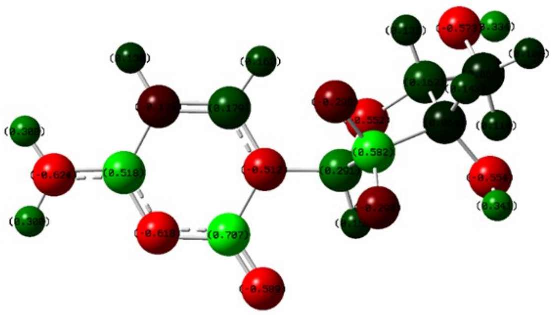

GEM-Drug

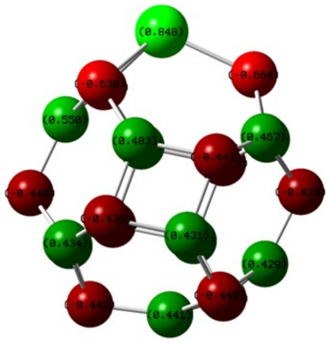

Fe-B11N12

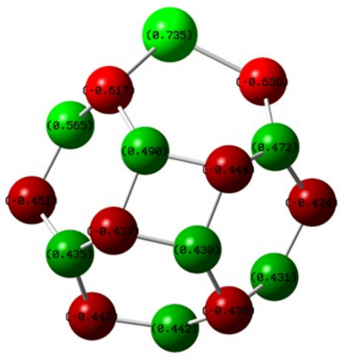

Co-B11N12

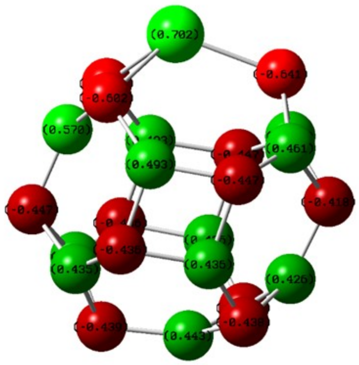

Ni-B11N12

Figure S2: NBO analysis of Drug an metal doped $B_{11}N_{24}$ before adsorption.

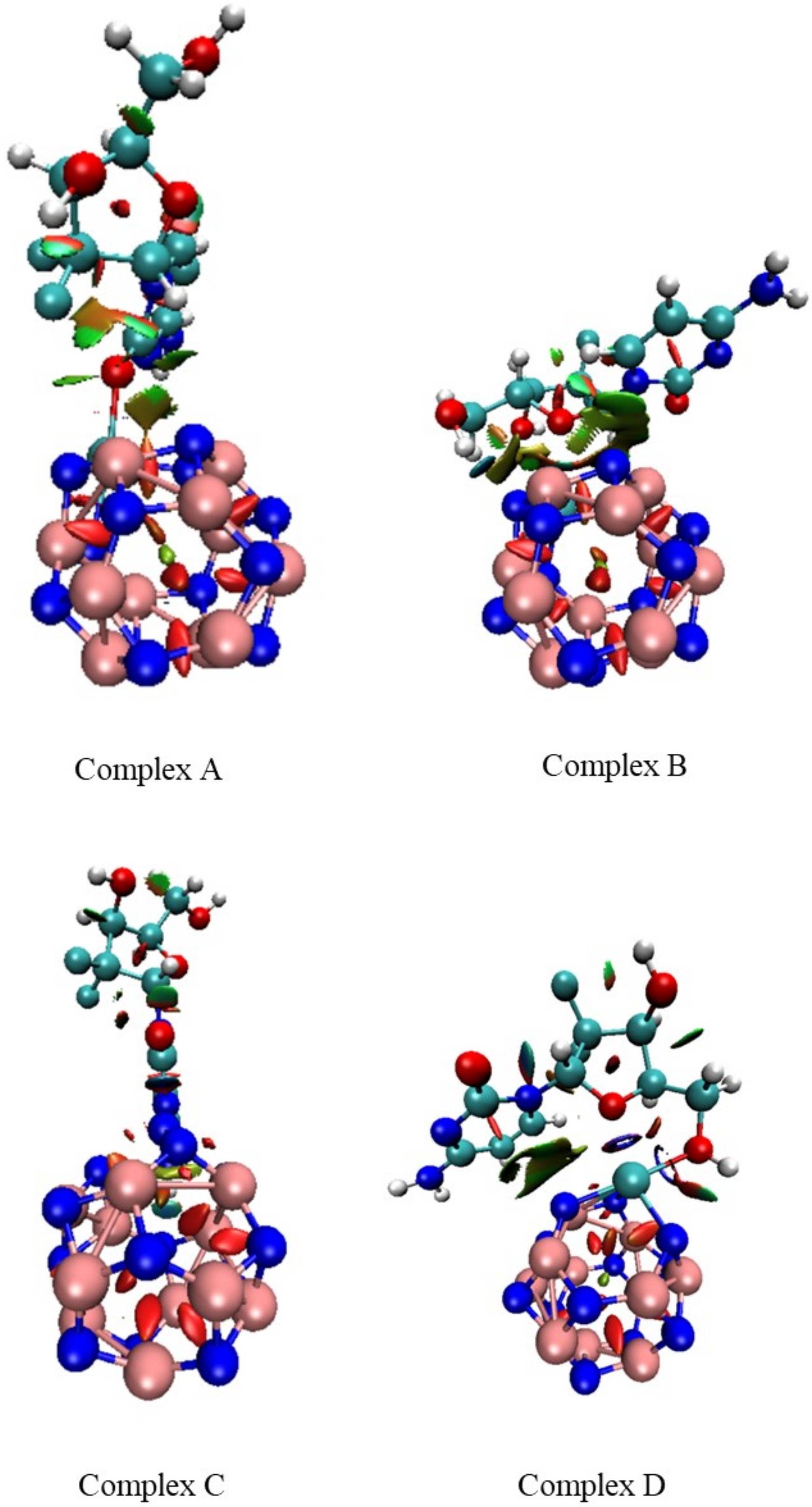

Figure S3: NCI analysis of investigated complexes.

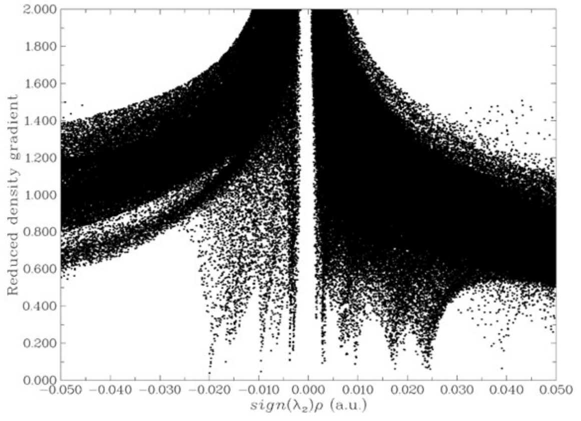

Complex A

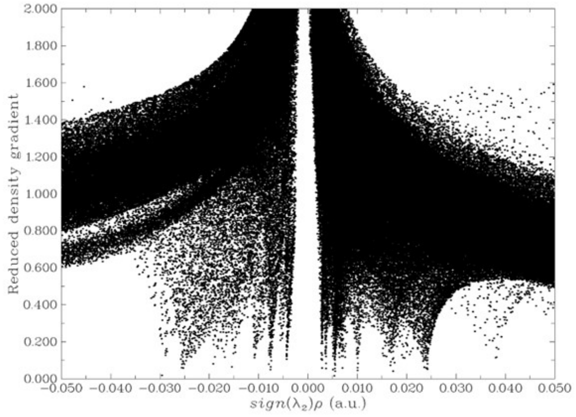

Complex B

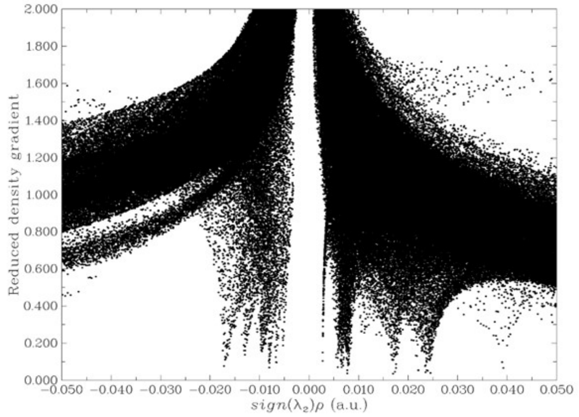

Complex C

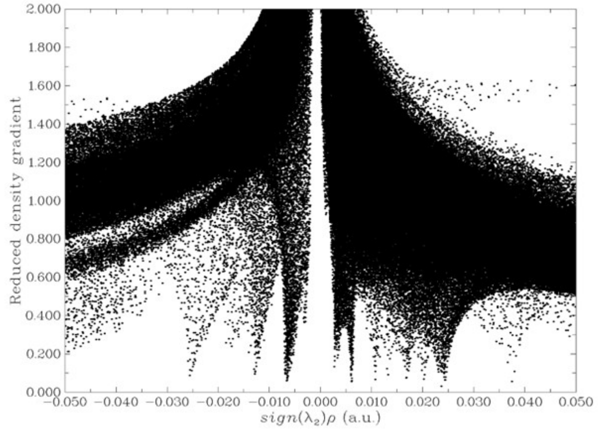

Complex D

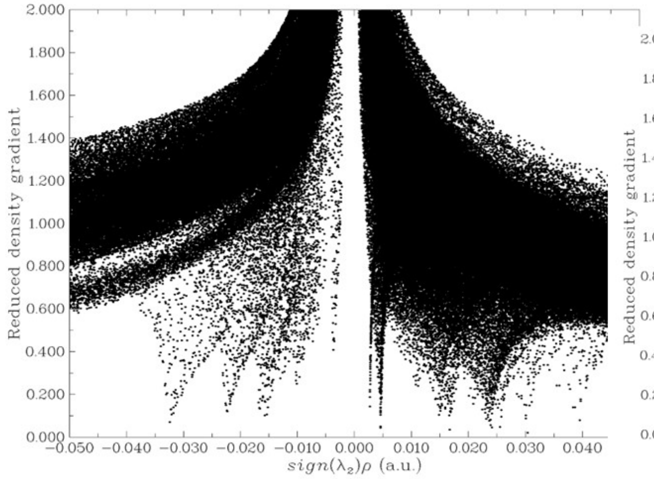

Complex E

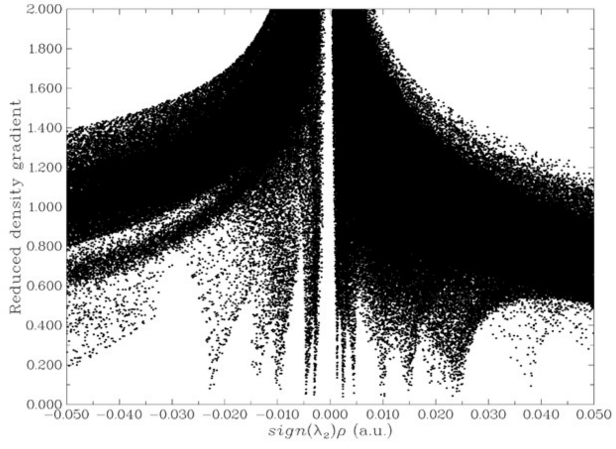

Complex F

Figure S4: RDG plots of GEM drug on interaction with metal doped $CoB_{11}N_{12}$, $FeB_{11}N_{12}$, and $NiB_{11}N_{12}$.

Table S1: Energy between donor and acceptor for complex A to complex F

	Donor NBO	Acceptor NBO	$E^{(2)}$ kcal/mol	$E_{(j)}$-$E_{(i)}$ a.u.	$F_{(i,j)}$ a.u.
Complex A	N_1-B_{20}	B_{23}	13.15	0.63	0.088
	N_1-B_{20}	Fe_{24}	11.03	0.87	0.088
	N_{11}-B_{13}	N_{12}-B_{18}	11.27	1.07	0.099
Complex B	N_1-B_{20}	Fe_{24}	9.20	0.89	0.082
	N_{11} Fe_{24}	$N_{12}Fe_{24}$	19.12	0.077	0.077
	N_2-B_{19}	B_{23}	3.68	1.32	0.063
Complex C	N_1-B_{17}	B_{23}	8.51	0.56	0.067
	N_1-Co_{24}	N_2- Co_{24}	15.92	0.39	0.073
	N_1-Co_{24}	N_3-B_{23}	5.55	0.63	0.055
Complex D	N_1-B_{17}	Co_{24}	16.25	0.70	0.096
	N_8	B_{14}	19.37	0.32	0.072
	N_8	B_{19}	11.61	0.33	0.057
Complex E	N_1	Ni_{24}	21.74	0.54	0.143
	N_8	B_{14}	8.00	0.35	0.068
	N_{12-} Ni_{24}	Ni_{24}	20.06	0.24	0.134
Complex F	O_{50}	Ni_{28-} C_{29}	14.62	0.54	0.113
	O_{32}	Ni_{24}	17.08	0.84	17.08
	N_6	B_{17}	10.10	0.33	0.075

Compelx A

Center Number	Atomic Number	Atomic Type	Coordinates (Angstroms)		
			X	Y	Z
1	7	0	-1.384541	1.502016	-0.677166
2	7	0	-2.463538	0.535148	-2.378489
3	7	0	-3.994889	1.813230	-0.719590
4	7	0	-2.948835	1.153966	1.588842
5	7	0	-5.225604	0.426847	0.546036
6	7	0	-3.801735	-1.277485	1.873198
7	7	0	-4.793908	-2.094259	0.024807
8	7	0	-2.234507	-2.548652	0.235838
9	7	0	-3.252498	-1.918466	-2.071380
10	7	0	-4.857972	-0.347067	-1.906797
11	7	0	-1.011643	-1.109350	-0.985547
12	7	0	-1.348325	-0.521397	1.585532
13	5	0	-2.150583	-2.055990	-1.157025
14	5	0	-3.531684	-2.250054	0.799617
15	5	0	-4.881669	-0.908884	0.933440
16	5	0	-4.023546	1.333786	0.701017

17	5	0	-2.771091	-0.233088	1.889474
18	5	0	-1.308468	-1.404949	0.454691
19	5	0	-3.469242	-0.487083	-2.399391
20	5	0	-1.356556	0.231563	-1.434962
21	5	0	-4.517222	-1.668724	-1.317184
22	5	0	-4.926190	0.669996	-0.875372
23	5	0	-2.718868	1.543659	-1.309559
24	26	0	-1.000508	1.192121	1.064339
25	6	0	2.664787	3.719792	-0.206899
26	6	0	3.840384	3.129980	-0.772208
27	6	0	3.902961	1.770972	-0.785427
28	7	0	2.889245	1.027123	-0.264371
29	6	0	1.759403	1.653173	0.271970
30	7	0	1.669310	2.986776	0.293258
31	6	0	2.902498	-0.462385	-0.315240
32	8	0	4.020426	-0.897940	-1.031667
33	6	0	4.999979	-1.485656	-0.131324
34	6	0	4.153766	-2.148456	0.949465
35	6	0	3.028441	-1.107348	1.101968
36	1	0	1.978827	-0.788605	-0.799019
37	9	0	3.390424	-0.176509	2.029739
38	9	0	1.878099	-1.701105	1.513714
39	6	0	5.907731	-2.394017	-0.930756
40	1	0	5.605156	-0.687301	0.321599
41	8	0	6.738837	-1.556290	-1.722199
42	1	0	5.286103	-3.064883	-1.534174
43	1	0	6.493952	-3.008021	-0.229245
44	7	0	2.519362	5.057631	-0.155390
45	1	0	1.653785	5.430387	0.204982
46	1	0	3.196808	5.681863	-0.559409
47	8	0	3.648994	-3.367587	0.459182
48	1	0	4.691645	-2.259634	1.901037
49	1	0	2.877019	-3.606652	0.992113
50	8	0	0.879469	0.872019	0.723220
51	1	0	7.157853	-2.100962	-2.399565
52	1	0	4.641148	3.726535	-1.189859
53	1	0	4.720850	1.209027	-1.217651

Complex B

Center Number	Atomic Number	Atomic Type	Coordinates (Angstroms)

			X	Y	Z
1	7	0	0.385202	0.242341	-1.168891
2	7	0	1.537746	2.152156	-1.035112
3	7	0	2.689302	0.315756	-2.452730
4	7	0	2.283244	-1.893420	-1.102715
5	7	0	4.494507	-0.566028	-1.451384
6	7	0	3.910013	-1.559698	0.873389
7	7	0	4.913778	0.450468	0.910988
8	7	0	2.734264	0.242680	2.322092
9	7	0	3.189765	2.405077	0.951398
10	7	0	4.127700	1.976184	-1.047510
11	7	0	0.952209	1.096266	1.257172
12	7	0	1.325496	-1.512848	1.016945
13	5	0	2.324829	1.505587	1.664054
14	5	0	3.891967	-0.313664	1.674231
15	5	0	4.685872	-0.677835	-0.032989
16	5	0	3.102263	-0.960849	-1.816937
17	5	0	2.599939	-1.847218	0.329384
18	5	0	1.491643	-0.226440	1.650519
19	5	0	2.833418	2.435172	-0.489181
20	5	0	0.725542	1.268089	-0.160319
21	5	0	4.356392	1.668893	0.389127
22	5	0	3.951195	0.764910	-1.806760
23	5	0	1.528093	0.907736	-1.845242
24	26	0	0.545355	-1.472492	-0.668856
25	6	0	-3.223085	3.551597	0.680881
26	6	0	-2.751069	2.637785	1.684648
27	6	0	-2.508054	1.363345	1.282394
28	7	0	-2.721387	0.995071	-0.009296
29	6	0	-3.247904	1.921557	-0.983271
30	7	0	-3.441612	3.207688	-0.575886
31	6	0	-2.520458	-0.365526	-0.496182
32	8	0	-1.632652	-1.097106	0.352418
33	6	0	-2.392498	-2.124101	1.091097
34	6	0	-3.475470	-2.548044	0.108908
35	6	0	-3.835572	-1.199216	-0.528639
36	1	0	-2.105759	-0.290746	-1.499467
37	9	0	-4.808060	-0.593657	0.209948
38	9	0	-4.308156	-1.373487	-1.792038
39	6	0	-1.487681	-3.233259	1.608806
40	1	0	-2.860223	-1.630037	1.952062
41	8	0	-0.654423	-2.801340	2.657847
42	1	0	-0.930339	-3.671602	0.772351
43	1	0	-2.144763	-4.020339	1.999590
44	7	0	-3.480949	4.841471	1.020413

45	1	0	-3.702004	5.477950	0.269799
46	1	0	-3.170453	5.219222	1.899565
47	8	0	-2.927744	-3.431254	-0.844980
48	1	0	-4.352655	-2.971137	0.617412
49	1	0	-3.489831	-3.398180	-1.632694
50	8	0	-3.476446	1.487601	-2.104166
51	1	0	0.166176	-2.469343	2.250028
52	1	0	-2.571031	2.943720	2.707079
53	1	0	-2.106239	0.606073	1.943025

C0mplex C

Center Number	Atomic Number	Atomic Type	Coordinates (Angstroms)		
			X	**Y**	**Z**
1	7	0	2.110275	-2.150620	-0.345248
2	7	0	1.566220	-0.135328	-1.387742
3	7	0	3.823731	-1.217826	-2.054269
4	7	0	4.670808	-2.334396	0.128523
5	7	0	5.641234	-0.261483	-1.124871
6	7	0	5.522311	-0.280161	1.481016
7	7	0	4.998746	1.676433	0.496270
8	7	0	3.298606	0.817075	2.271086
9	7	0	2.433854	1.889757	0.063781
10	7	0	3.677381	1.349457	-1.725902
11	7	0	1.419696	-0.098084	1.454685
12	7	0	3.410141	-1.758173	1.897518
13	5	0	2.219727	1.102955	1.263701
14	5	0	4.600075	0.871445	1.684288
15	5	0	5.660991	0.388221	0.159449
16	5	0	4.860781	-1.531338	-1.047157
17	5	0	3.215691	-2.303940	0.522461
18	5	0	4.762957	-1.504139	1.350499
19	5	0	2.668020	-0.528892	2.073329
20	5	0	2.309794	1.054313	-1.160511
21	5	0	3.851150	1.889177	-0.356849
22	5	0	4.412655	0.125663	-1.881221
23	5	0	2.487820	-1.268624	-1.437078
24	27	0	0.653048	-0.975633	0.022007
25	6	0	-1.434140	3.198859	-0.285824
26	6	0	-2.856301	3.352622	-0.240022
27	6	0	-3.590664	2.229596	-0.031885
28	7	0	-2.986296	1.011923	0.114985
29	6	0	-1.597959	0.914691	0.022634
30	7	0	-0.851275	1.996943	-0.161351

31	6	0	-3.776109	-0.215720	0.351626
32	8	0	-5.029848	0.114433	0.887328
33	6	0	-6.115057	-0.450154	0.085517
34	6	0	-5.472084	-1.588278	-0.703243
35	6	0	-4.079390	-0.998978	-0.952087
36	1	0	-3.200455	-0.841243	1.035903
37	9	0	-4.116690	-0.138890	-2.014246
38	9	0	-3.153818	-1.957268	-1.226596
39	6	0	-7.252723	-0.855465	0.999144
40	1	0	-6.469489	0.318875	-0.611206
41	8	0	-7.832739	0.334986	1.516638
42	1	0	-6.862077	-1.502935	1.792379
43	1	0	-7.975072	-1.438079	0.408087
44	7	0	-0.619556	4.246616	-0.456036
45	1	0	0.378467	4.088126	-0.447738
46	1	0	-0.969258	5.188553	-0.533550
47	8	0	-5.367911	-2.729054	0.123313
48	1	0	-5.988981	-1.790362	-1.648467
49	1	0	-4.869179	-3.403586	-0.359949
50	8	0	-1.113724	-0.257870	0.118006
51	1	0	-8.499200	0.076055	2.166633
52	1	0	-3.333941	4.317408	-0.346240
53	1	0	-4.667649	2.232561	0.058428

Complex D

Center Number	Atomic Number	Atomic Type	Coordinates (Angstroms)		
			X	**Y**	**Z**
1	7	0	0.426061	0.553577	-0.706100
2	7	0	0.603312	-0.616238	1.302625
3	7	0	1.711388	1.724376	1.222261
4	7	0	2.574390	1.942046	-1.211091
5	7	0	3.953466	1.706469	0.990522
6	7	0	4.701739	0.446415	-1.164638
7	7	0	4.889936	-0.715323	0.755202
8	7	0	3.681981	-1.942358	-1.191594
9	7	0	2.756002	-2.145197	1.227414
10	7	0	2.930857	-0.236560	2.398458
11	7	0	1.442151	-2.058736	-1.042325
12	7	0	2.410829	0.008359	-2.338496
13	5	0	2.623275	-2.295624	-0.205329
14	5	0	4.579167	-0.951935	-0.679818

15	5	0	4.704254	0.690228	0.302056
16	5	0	2.735910	2.065474	0.208519
17	5	0	1.540443	0.893290	-1.517459
18	5	0	3.454972	0.882363	-1.749759
19	5	0	2.466511	-1.327819	-1.802202
20	5	0	1.867073	-1.084706	1.767616
21	5	0	3.781939	-1.130357	1.579685
22	5	0	2.895439	1.085960	1.836769
23	5	0	0.801728	0.700407	0.690911
24	27	0	-0.060296	-1.240866	-0.343755
25	6	0	-1.434012	3.768421	0.192662
26	6	0	-1.577085	2.926030	1.354007
27	6	0	-2.185500	1.733774	1.171396
28	7	0	-2.678891	1.381708	-0.060427
29	6	0	-2.519326	2.265026	-1.216953
30	7	0	-1.900562	3.455411	-1.000734
31	6	0	-3.138612	0.064067	-0.336562
32	8	0	-2.125957	-0.941973	-0.009610
33	6	0	-2.718023	-2.111958	0.630486
34	6	0	-4.226824	-1.968673	0.431614
35	6	0	-4.368869	-0.442063	0.434051
36	1	0	-3.352759	0.038402	-1.406788
37	9	0	-4.384800	0.001740	1.727995
38	9	0	-5.527081	-0.042749	-0.155654
39	6	0	-2.076872	-3.333283	-0.000509
40	1	0	-2.467508	-2.065359	1.695969
41	8	0	-0.661974	-3.109235	0.139396
42	1	0	-2.365412	-3.412701	-1.051733
43	1	0	-2.368286	-4.244156	0.532579
44	7	0	-0.803793	4.962028	0.321040
45	1	0	-0.586503	5.448719	-0.536752
46	1	0	-0.218028	5.136363	1.121919
47	8	0	-4.585282	-2.493413	-0.826789
48	1	0	-4.797579	-2.413313	1.257530
49	1	0	-5.463267	-2.155075	-1.055469
50	8	0	-2.955678	1.882412	-2.291080
51	1	0	-0.125003	-3.680342	-0.436360
52	1	0	-1.170734	3.202797	2.317650
53	1	0	-2.293755	1.008510	1.965508

Complex E

Center Number	Atomic Number	Atomic Type	Coordinates (Angstroms)		
			X	**Y**	**Z**

1	7	0	1.514060	-0.145806	-1.402436
2	7	0	3.541177	0.060546	-2.359127
3	7	0	3.133395	-2.186994	-1.130482
4	7	0	1.947541	-1.462002	1.112682
5	7	0	4.346151	-2.389649	0.748180
6	7	0	3.933294	-0.480417	2.450094
7	7	0	5.822871	-0.297776	1.237318
8	7	0	4.269403	1.794602	1.251778
9	7	0	5.452673	1.086942	-0.942638
10	7	0	5.529012	-1.148343	-1.207549
11	7	0	2.981113	1.902491	-0.595672
12	7	0	1.877839	0.820588	1.563844
13	5	0	4.427317	1.785452	-0.215438
14	5	0	4.768170	0.569066	1.826761
15	5	0	4.796495	-1.320858	1.594972
16	5	0	2.923607	-2.158560	0.333451
17	5	0	2.572800	-0.414255	1.917558
18	5	0	2.839724	1.603536	0.874350
19	5	0	4.850375	0.032663	-1.801539
20	5	0	2.577920	0.834308	-1.522852
21	5	0	5.881489	-0.126497	-0.188920
22	5	0	4.541225	-2.063907	-0.684128
23	5	0	2.638458	-0.988728	-1.779483
24	28	0	0.775729	-0.215070	0.366121
25	6	0	-0.905709	2.303558	-0.446980
26	6	0	-2.244905	2.793684	-0.625382
27	6	0	-3.276758	1.952327	-0.368964
28	7	0	-3.057754	0.671478	0.056900
29	6	0	-1.753869	0.204215	0.239440
30	7	0	-0.714404	1.054456	-0.004734
31	6	0	-4.165009	-0.258769	0.297696
32	8	0	-5.288945	0.448868	0.759383
33	6	0	-6.506088	-0.011139	0.097886
34	6	0	-6.113969	-1.281567	-0.661375
35	6	0	-4.651044	-0.972986	-0.980692
36	1	0	-3.805281	-0.999286	1.016077
37	9	0	-4.582729	-0.118357	-2.047902
38	9	0	-3.928544	-2.081306	-1.280587
39	6	0	-7.610132	-0.188500	1.122045
40	1	0	-6.812271	0.761715	-0.617584
41	8	0	-8.005500	1.108746	1.537586
42	1	0	-7.229174	-0.799744	1.948177
43	1	0	-8.438981	-0.738429	0.647943
44	7	0	0.143486	3.087868	-0.704061
45	1	0	1.106168	2.753372	-0.640093

46	1	0	-0.007447	4.017374	-1.061523
47	8	0	-6.188816	-2.391250	0.208722
48	1	0	-6.700954	-1.418347	-1.579178
49	1	0	-5.730940	-3.133732	-0.211120
50	8	0	-1.513416	-0.950803	0.612081
51	1	0	-8.546387	1.019303	2.331615
52	1	0	-2.426932	3.808772	-0.952847
53	1	0	-4.315330	2.241511	-0.453007

Complex F

Center Number	Atomic Number	Atomic Type	Coordinates (Angstroms)		
			X	**Y**	**Z**
1	7	0	-0.619638	-1.389700	1.024918
2	7	0	-2.344502	-0.850656	2.378358
3	7	0	-3.008431	-2.222841	0.276981
4	7	0	-1.756212	-1.217303	-1.830182
5	7	0	-4.271827	-1.077530	-1.191430
6	7	0	-3.025269	1.025913	-2.061505
7	7	0	-4.580783	1.409815	-0.479502
8	7	0	-2.217439	2.413266	-0.026622
9	7	0	-3.590940	1.382388	1.930325
10	7	0	-4.677562	-0.487390	1.306324
11	7	0	-1.039188	1.169721	1.425464
12	7	0	-0.627340	0.680486	-1.142338
13	5	0	-2.358379	1.837915	1.336260
14	5	0	-3.244878	1.917021	-0.899908
15	5	0	-4.176049	0.339436	-1.429375
16	5	0	-2.900923	-1.661422	-1.092033
17	5	0	-1.812760	0.241449	-1.878708
18	5	0	-1.056802	1.466509	-0.016806
19	5	0	-3.523363	-0.086531	2.155464
20	5	0	-1.119183	-0.242474	1.767714
21	5	0	-4.534306	0.929521	0.876946
22	5	0	-4.231382	-1.370643	0.260726
23	5	0	-2.010817	-1.759880	1.241124
24	28	0	-0.140142	-1.110077	-0.855758
25	6	0	2.385804	3.798081	-0.433083
26	6	0	2.669880	2.929254	-1.545827
27	6	0	2.786534	1.608927	-1.272081
28	7	0	2.666695	1.151816	0.011939
29	6	0	2.451672	2.066952	1.129804

30	7	0	2.281277	3.379998	0.815053
31	6	0	2.745525	-0.239250	0.334602
32	8	0	1.992314	-1.034903	-0.617588
33	6	0	2.558614	-2.386777	-0.708892
34	6	0	3.838722	-2.386701	0.143081
35	6	0	4.133773	-0.886481	0.238103
36	1	0	2.360945	-0.356190	1.350021
37	9	0	4.780050	-0.479801	-0.897289
38	9	0	4.922612	-0.590228	1.301689
39	6	0	1.492136	-3.367388	-0.260194
40	1	0	2.796947	-2.541264	-1.765416
41	8	0	0.347584	-3.063631	-1.092054
42	1	0	1.237901	-3.209064	0.788451
43	1	0	1.815694	-4.398308	-0.433375
44	7	0	2.230677	5.127785	-0.653083
45	1	0	1.902797	5.681417	0.124604
46	1	0	2.086152	5.480603	-1.584704
47	8	0	3.558003	-2.915790	1.417879
48	1	0	4.661305	-2.912152	-0.359807
49	1	0	4.261613	-2.639013	2.022987
50	8	0	2.441977	1.598418	2.255779
51	1	0	-0.421742	-3.572208	-0.791049
52	1	0	2.753602	3.299010	-2.559536
53	1	0	2.957385	0.862238	-2.035693
